# *Trans*-Golgi network localized small GTPase RabA1d is involved in cell plate formation and oscillatory root hair growth

**DOI:** 10.1186/s12870-014-0252-0

**Published:** 2014-09-27

**Authors:** Tobias Berson, Daniel von Wangenheim, Tomáš Takáč, Olga Šamajová, Amparo Rosero, Miroslav Ovečka, George Komis, Ernst HK Stelzer, Jozef Šamaj

**Affiliations:** Institute of Cellular and Molecular Botany, University of Bonn, Kirschallee 1, Bonn, D-53115 Germany; Centre of the Region Haná for Biotechnological and Agricultural Research, Department of Cell Biology, Faculty of Science, Palacký University, Šlechtitelů 11, Olomouc, 783 71 Czech Republic; Buchmann Institute for Molecular Life Sciences, Goethe-Universität Frankfurt am Main, Max-von-Laue-Str. 15, Frankfurt am Main, 60438 Germany

**Keywords:** *Arabidopsis*, Cell plate, Cytokinesis, Rab GTPase, Localization, RabA1d, Root hair, *Trans*-Golgi network, Vesicle

## Abstract

**Background:**

Small Rab GTPases are important regulators of vesicular trafficking in plants. AtRabA1d, a member of the RabA1 subfamily of small GTPases, was previously found in the vesicle-rich apical dome of growing root hairs suggesting a role during tip growth; however, its specific intracellular localization and role in plants has not been well described.

**Results:**

The transient expression of *35S*::*GFP*:*RabA1d* construct in *Allium porrum* and *Nicotiana benthamiana* revealed vesicular structures, which were further corroborated in stable transformed *Arabidopsis thaliana* plants. GFP-RabA1d colocalized with the *trans*-Golgi network marker mCherry-VTI12 and with early FM4-64-labeled endosomal compartments. Late endosomes and endoplasmic reticulum labeled with FYVE-DsRed and ER-DsRed, respectively, were devoid of GFP-RabA1d. The accumulation of GFP-RabA1d in the core of brefeldin A (BFA)-induced-compartments and the quantitative upregulation of RabA1d protein levels after BFA treatment confirmed the association of RabA1d with early endosomes/TGN and its role in vesicle trafficking. Light-sheet microscopy revealed involvement of RabA1d in root development. In root cells, GFP-RabA1d followed cell plate expansion consistently with cytokinesis-related vesicular trafficking and membrane recycling. GFP-RabA1d accumulated in disc-like structures of nascent cell plates, which progressively evolved to marginal ring-like structures of the growing cell plates. During root hair growth and development, GFP-RabA1d was enriched at root hair bulges and at the apical dome of vigorously elongating root hairs. Importantly, GFP-RabA1d signal intensity exhibited an oscillatory behavior in-phase with tip growth. Progressively, this tip localization dissapeared in mature root hairs suggesting a link between tip localization of RabA1d and root hair elongation. Our results support a RabA1d role in events that require vigorous membrane trafficking.

**Conclusions:**

RabA1d is located in early endosomes/TGN and is involved in vesicle trafficking. RabA1d participates in both cell plate formation and root hair oscillatory tip growth. The specific GFP-RabA1d subcellular localization confirms a correlation between its specific spatio-temporal accumulation and local vesicle trafficking requirements during cell plate and root hair formation.

**Electronic supplementary material:**

The online version of this article (doi:10.1186/s12870-014-0252-0) contains supplementary material, which is available to authorized users.

## Background

Cell expansion and division are fundamental processes in all organisms. They require a constant supply of proteins and lipids to generate a new plasma membrane and cell wall, and also the retrieval of excess membrane and recycling protein fractions by endocytotic processes which allow to maintain the membrane homeostasis in the cell [1-3]. These highly dynamic events are orchestrated by several regulators of vesicle trafficking. Among such factors, small GTPases of the Rab family hold a central role in plant cells [4-6]. Rab GTPases are members of the Ras small GTPase superfamily. Rab GTPases cycle between an active GTP-bound and membrane associated form and an inactive, GDP-bound and predominantly cytosolic form, and are involved in membrane identity and specificity of vesicle targeting during vesicular trafficking, tethering and fusion [7]. Mechanistically, Rab GTPases execute the kinetic proofreading of specific membrane surfaces via their reversible and GTP/GDP-dependent association/dissociation with membranes. In their active, GTP-bound form, Rab GTPases are engaged into indirect interactions with coat components, motor proteins and SNAREs. Thus, Rab GTPases emerged as multifaceted organizers in membrane trafficking processes in eukaryotic cells [8-10]. The family of Rab GTPases is particularly expanded in the *Arabidopsis* genome with 57 individual members classified in 8 subfamilies (RabA to RabH). The most extensive subfamily in plants (corresponding to the *Arabidopsis* genome) is RabA (Rab11) encompassing 26 of the total 57 identified *Arabidopsis* Rabs [4,7,11].

Bearing in mind that by comparison to *Arabidopsis*, mammalian (with only three members; Rab11a, Rab11b and Rab25/Rab11c [12]) and yeast (with only two members; Ypt31/32 [13,7]) RabA homologues are much less represented [14], it is a question to understand the specific functions of individual RabAs in plants. In this sense, it is important to survey plant RabA diversity in terms of protein – protein interactions, specificity in intracellular localization patterns and possible differential developmental regulation at the tissue and organ level.

Previous studies have already illustrated the polar localization of some RabAs (RabA4d [15], RabA4b [16], RabA2 [17], RabA1d [18]) in tip-growing cells, and its significance in tip-targeted cell wall deposition during elongation of pollen tubes and root hairs. The role of RabA4b in polarized vesicular secretion of cell wall material is associated with the activity of effector protein PI-4Kβ1 and can be affected by disruption of actin microfilaments [16,19].

The role of plant RabAs is not restricted to polar growth but is also associated with cytokinesis. In plants, the spatially and temporally-controlled post-mitotic partitioning of daughter cells through the cytokinetic deposition of cell plate is a process which is heavily dependent on membrane trafficking and endocytosis [20-23]. Starting with the preprophase definition of cell division plane, where the microtubule preprophase band marks a site of intensive clathrin-mediated membrane retrieval [22,23], the cytokinetic process of cell plate deposition is robustly associated with endocytotic-related deposition of wall material by cell plate targeting of vesicles identified through the Rab5 *Arabidopsis* orthologue Ara7 [20]. Cytokinetic progression in *Arabidopsis* also recruited RabA2, RabA3 and RabA1c which colocalized with FM4-64 and partially with vacuolar H^+^-ATPase subunit a1 (VHA-a1) in early endosomes and TGN [24,25]. The relative contribution of endocytosis during cell plate formation is not completely understood, however, several observations suggest its essential role. Cell surface materials and exogenously applied endocytic tracers were rapidly delivered to the forming cell plate [20,26], while the KNOLLE syntaxin localized to endosomes previous to cell plate initiation and its localization in the plane of cell division involves endocytotic-related proteins [20,27,28]. Some of these proteins use a clathrin-mediated mechanism [29,30] and their mutations confirm *in vivo* the role in cytokinesis [24,30]. Similarly, other Rab-GTPases showed to be involved in endocytotic processes, such as RabF2a, RabF2b and RabF1 which are activated by VPS9a [31] and are localized in both early but preferentially in late/multivesicular endosomes [32-34].

The role of Rab GTPases is not restricted to endocytosis but has been also suggested in secretory trafficking (e.g., for RabD1 and RabD2; [35]). Secretory roles may be also attributed to RabA subfamily members since some of them were reported to localize in specific TGN compartments at the nexus of endocytosis and secretion [26]. Such TGN compartments were further corroborated by their aggregation following treatment with concanamycin A, an inhibitor of vacuolar H^+^−ATPases [36] and their insensitivity to wortmannin (a potent and specific inhibitor of phosphoinositide-3-kinase and inhibitor of vacuolar transport; [24]). Moreover, RabA2a and VHA-a1 are mislocalized in the *echidna* (*ech*) mutant of *Arabidopsis thaliana*, whereby endocytosis is unaffected but secretory trafficking is impaired [36]. Finally, the remarkable colocalizations of RabA1b with VAMP721, R- soluble N-ethyl-maleimide sensitive factor attachment protein receptor (R-SNARE), but partial and unstable associations with TGN, Golgi and endosome markers suggest a specific role in transport between TGN and the plasma membrane [37].

Our study reports the subcellular localization of RabA1d and its role in vesicle trafficking. Using light-sheet microscopy ensuring cell viability and stress-free root development during imaging, we found that RabA1d is involved in two essential cellular processes, cell plate expansion during cytokinesis and oscillatory tip growth in root hairs, representing highly active vesicle trafficking events.

## Results

### GFP-RabA1d localizes to vesicle-like structures at *trans*-Golgi network compartment

**Figure 1:**
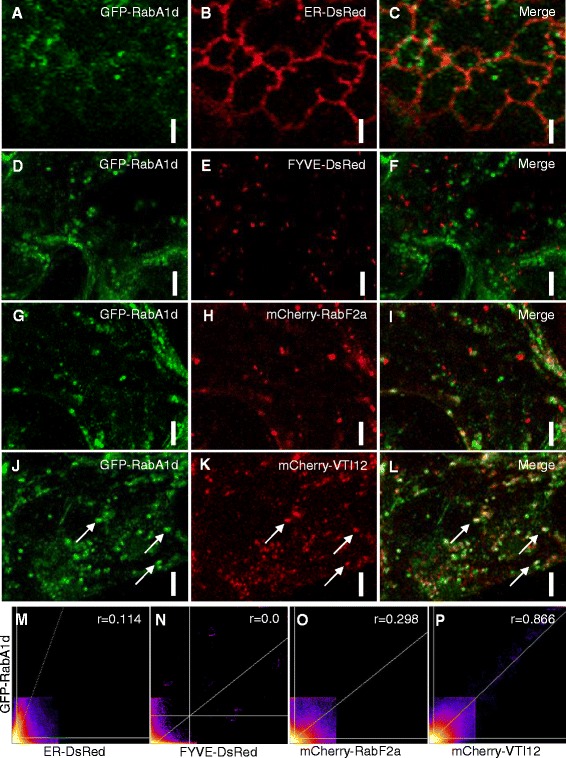
**Subcellular localization of GFP**-**tagged RabA1d.** Subcellular localization of GFP-RabA1d in cells of *N. benthamiana*. Co-vizualization with ER reporter ER-DsRed **(A**-**C)** showed partial association of GFP-RabA1d and cortical ER tubules. GFP-RabA1d colocalized with neither 2xFYVE-DsRed **(D**-**F)** nor with mCherry-RabF2a **(G**-**I)**, markers for late endosomes/multi-vesicular bodies. Colocalization of GFP-RabA1d with mCherry-VTI12 representing a TGN marker **(J**-**L)**. Intensity correlation scatterplots of GFP-Rab1Ad and ER-DsRed **(M)**, FYVE-DsRed **(N)**, mCherry-RabF2a **(O)**, and mCherry-VTI12 **(P)**. Pearson’s coefficient (r) was determined after Costes automatic threshold. Bars represent 3 μm in A-C and 5 μm in D-L.

In order to follow the localization patterns of RabA1d, we engineered a GFP-RabA1d expressed under cauliflower mosaic virus *35S* promoter. Specificity of GFP-RabA1d localization was tested by transient expression of *35S*::*GFP*:*RabA1d* construct in *Allium porrum* and *Nicotiana benthamiana* (Figure 1A,D,G,J; Additional file 1: Figure S1A,B) and was confirmed in seedlings of *Arabidopsis thaliana* stably transformed with the same construct (Additional file 1: Figure S1C). The expression of the fusion protein was verified by western blotting with a monoclonal antibody against GFP showing a single band at ca. 46 kDa, corresponding to the molecular weight of the GFP-RabA1d fusion (Additional file 1: Figure S1D).

In both cases of transient transformation, GFP-RabA1d localized to spot-like structures that were rapidly moving through the cytoplasm (Additional file 1: Figure S1A,B). Moreover, the localization of GFP-RabA1d was cross-compared with endomembrane markers of known specificity including endoplasmic reticulum (HDEL signal peptide fused to DsRed [38]; designated ER-DsRed), late endosomes and prevacuolar compartments (double FYVE domain from mouse Hrs protein fused to DsRed; designated FYVE-DsRed [39,40] and mCherry-RabF2a [4]) and *trans*-Golgi network (TGN)/early endosomes (VTI12 SNARE protein fused to mCherry; designated mCherry-VTI12 [34,41,42]). GFP-RabA1d did not colocalize with ER-DsRed, however, motile spot-like structures containing GFP-RabA1d appeared in close proximity to ER tubules (Figure 1A-C,M). The observation of the co-expression of FYVE-DsRed and mCherry-RabF2a with GFP-RabA1d revealed no significant colocalization with the late endosomal markers (Figure 1D-I,N,O). Interestingly, an obvious colocalization was observed for GFP-RabA1d and mCherry-VTI12, a TGN marker. This colocalization showed good quantitative correlation; thereby, RabA1d is localized at the TGN compartments (Figure 1J-L,P).

### GFP-RabA1d accumulates in BFA compartments and is upregulated by BFA treatment

The above results indicated a localization of GFP-RabA1d in TGN/early endosomes as expected for many other members of RabA class. To better substantiate this localization pattern, *Arabidopsis* seedlings stably expressing the GFP-RabA1d fusion were co-stained with the membrane/endocytotic tracer FM4-64 [43], which depending on the immediacy of microscopic observation, localizes fully or partially with early endosomes such as those labeled with fluorescent protein-tagged VTI12 (e.g. [34]).

**Figure 2:**
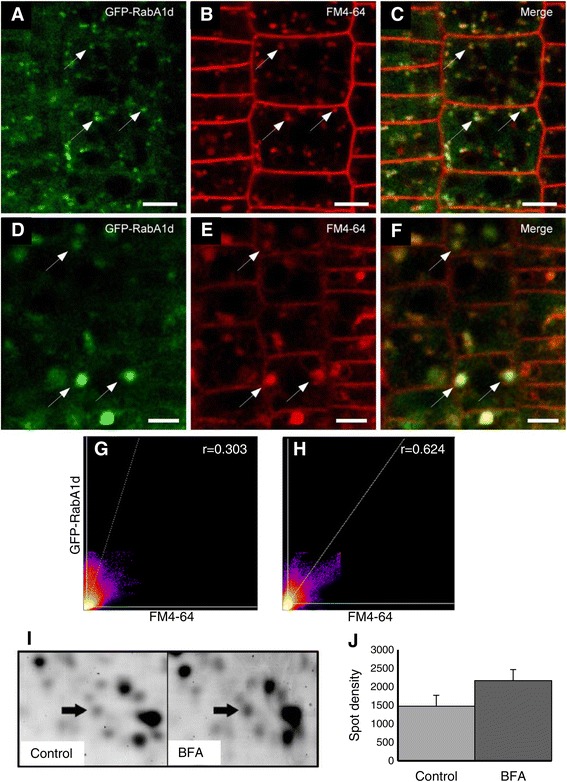
**GFP**-**RabA1d accumulates in BFA compartments and is upregulated by BFA treatment.** Root cells of *Arabidopsis* stably transformed with *35S*::*GFP*:*RabA1d* construct were analysed. GFP-RabA1d colocalized with early endocytotic compartments labeled by FM4-64 **(A**-**C)**. After BFA treatment, both GFP-RabA1d and FM4-64 accumulated together in the core of BFA compartments **(D**-**F)**. 2D-histogram intensity and correlation of GFP-Rab1Ad and FM4-64 early endocytotic compartments in root cells **(G)** and after BFA treatment **(H)**. Pearson’s coefficient (r) was determined using Costes automatic threshold. BFA treatment induced RabA1d upregulation at protein level **(I)**, upregulation of RabA1d was determined from comparison of 2-DE gels (arrow) and measured as increase of spot density **(J)**. Bars represent 4 μm in A-C and 5 μm in D-F.

In this case, the GFP-RabA1d vesicles colocalized with early FM4-64 compartments of the endocytotic pathway within 6–15 min after application of the dye (Figure 2A-C). It was additionally confirmed by comparison with YFP-RabF2a late endosomal marker which showed partial colocalization with FM4-64 compartments only after 15 min (Additional file 1: Figure S2A,B). Next, FM4-64 stained roots were treated with BFA, a fungal toxin that inhibits exocytosis and endocytotic recycling without affecting the first steps of endocytosis [44,45]. Importantly, after treatment with BFA, GFP-RabA1d relocalized and accumulated in the core of BFA-compartments along with FM4-64 (Figure 2D-F). These BFA-compartments are composed of TGN and plasma membrane-derived endocytotic vesicles in the core, surrounded by remnants of Golgi stacks [44]. The colocalization of GFP-RabA1d and FM4-64 showed good quantitative correlation and it was increased after BFA-treatment (Figure 2G,H). After BFA washout, the GFP-RabA1d and FM4-64 compartments started to deliberate from BFA compartments within 5 min and progressively redistributed in the root cells. Importantly, both GFP-RabA1d and FM4-64 compartments remained colocalized during the release from the BFA compartments (Additional file 1: Figure S3A-E).

A proteomic analysis of BFA-treated *Arabidopsis* roots, showed the quantitative upregulation of RabA1d protein levels. This induction reached 1.35 fold (Figure 2I,J), however it slightly exceeded the significance level (P = 0.061). RabA1d identity was confirmed by a MOWSE score of 60 and 25% sequence coverage with 7 peptides matching (Additional file 1: Figure S4A,B). Therefore, RabA1d is involved in vesicle trafficking, its expression and localization in TGN/early endosomes is affected by BFA.

### GFP-RabA1d accumulates in growing cell plates during cytokinesis

**Figure 3:**
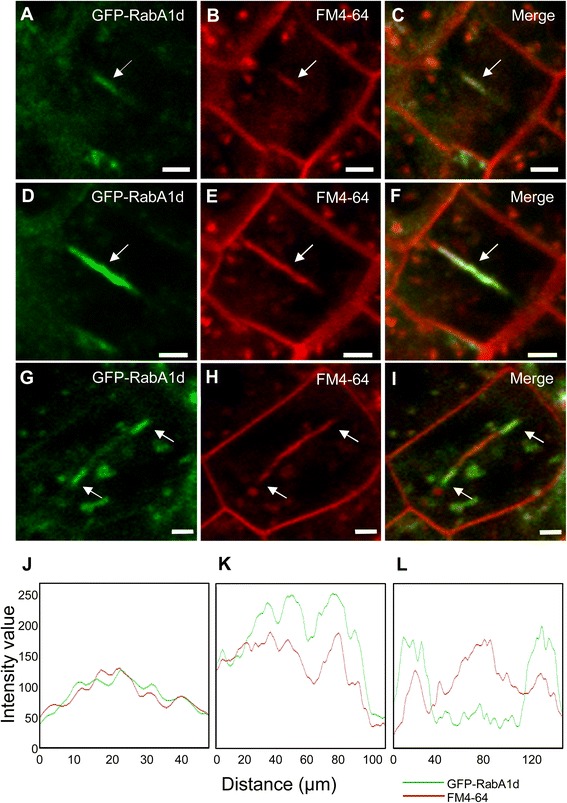
**Localization of GFP**-**RabA1d during the cell plate formation in cytokinetic cells.** During cell plate initiation, GFP-RabA1d localized in the mid plane of the dividing cell and completely colocalized with FM4-64 **(A**-**C)**. In the early stage of growing cell plates, GFP-RabA1d still appeared in the mid-plane of the cell **(D**-**F)**. During cytokinesis, GFP-RabA1d was accumulated mainly at the edges of growing cell plate **(G**-**I)**. Intensity profile of GFP-RabA1d and FM4-64 signals during cell plate initiation and early stage of plate expansion **(J**,**K)**, the two channels showed similar intensity and distribution; while in growing cell plate, GFP-RabA1d showed more intensity at the edges of growing cell plate than FM4-64 **(L)**. Bars represent 2 μm.

In dividing root meristematic cells, GFP-RabA1d specifically accumulated at the plane of cell plate deposition following similar centrifugal expansion observed for the microtubular phragmoplast. Thus, at the onset of cytokinesis and during the early stages of cell plate formation, GFP-RabA1d uniformly labeled the entire cell plate forming a thin continuous line when visualized cross-sectioned and fully co-localized with the FM4-64 marker (Figure 3A-F,J,K). At more advanced stages, GFP-RabA1d signal was restricted to the margins of the growing cell plate unmixing from FM4-64 labeling at the central region of the cell plate (Figure 3G-I,L).

**Figure 4:**
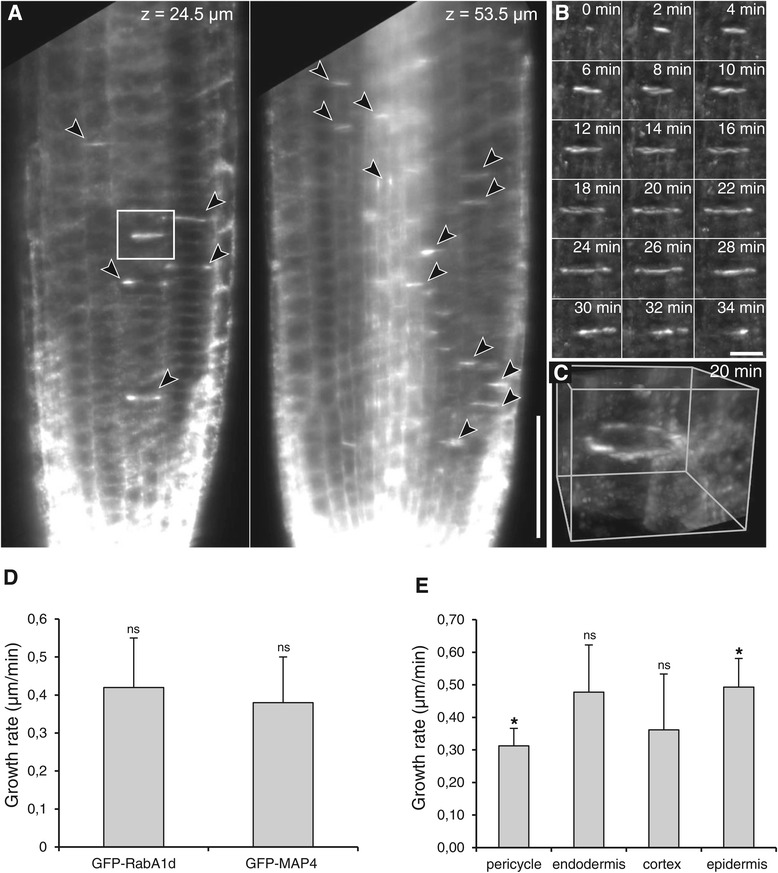
**Light**-**sheet live imaging of GFP**-**RabA1d accumulation during cell plate formation in the root meristem.** Meristematic cells undergoing cytokinesis showed several locations of GFP-RabA1d accumulation observed in single sections of one time point at indicated depths inside the primary root **(A)**, GFP-RabA1d accumulation in cell plates (arrowheads) of cytokinetic cells. The boxed area in **A** is enlarged shown in **B**. Detailed time-lapse imaging of GFP-RabA1d accumulation during single cell plate formation starting from spot like structures in the middle of the cell, followed by increased intensity during vesicle fusion and later formation of a ring-like structure at the margins in the growing cell plates **(B)**. Three-dimensional reconstruction of the cell plate shown in **(B)** at time point 20 min **(C)**. Comparison of cell plate growth rates in *Arabidopsis* plants stably transformed with in *35S*::*GFP*:*RabA1d* and *35S*::*GFP*:*MAP4* constructs (ns: no significant difference) **(D)**. Growth rates of cell plates in cells from different tissues **(E)**. *significant difference from Wilcoxon rank sum test with Holm’s correction (p<0.05), ns: no significant difference. Bars represent 50 μm in A and 10 μm in B.

The cytokinesis-related localization patterns of GFP-RabA1d were further followed by light-sheet microscopy which allows the observation of a physiological, unstressed root growth [46]. The cells undergoing cytokinesis in the meristem showed several locations of GFP-RabA1d accumulation (Figure 4A). The accumulation patterns of GFP-RabA1d coincided with areas of active vesicle fusion during cell plate formation and growth. In the initiating young cell plates, disc-like structures were observed, starting from diffuse expression probably during accumulation of vesicles and increased intensity during fusion of vesicles (Figure 4B, Additional file 1: Figure S5A,B). During cell plate expansion, it formed a ring-like structure at the margins of the growing cell plates (Figure 4B,C, Additional file 1: Figure S5A,B, Additional file 2: Video S1). The localization in the growing cell plate of GFP-RabA1d during cytokinesis suggests a role in delivery of membranes/cargo for cell plate formation and membrane recycling at margin domains (Additional file 1: Figure S5C). The growth rate of cell plates labeled with GFP-Rab1Ad was comparable to that observed with GFP-MAP4 (Figure 4D, Additional file 3: Video S2). However, significant differences were found between epidermis and pericycle cells expressing GFP-Rab1Ad (Figure 4E).

It is important to note that the overexpression of GFP-RabA1d had no serious consequences for plant growth and gravitropic response when transgenic plants were compared with non-transformed wild type plants (Additional file 1: Figure S6A-E).

### Oscillatory accumulation of GFP-RabA1d in tips of growing root hairs correlates with growth rate

**Figure 5:**
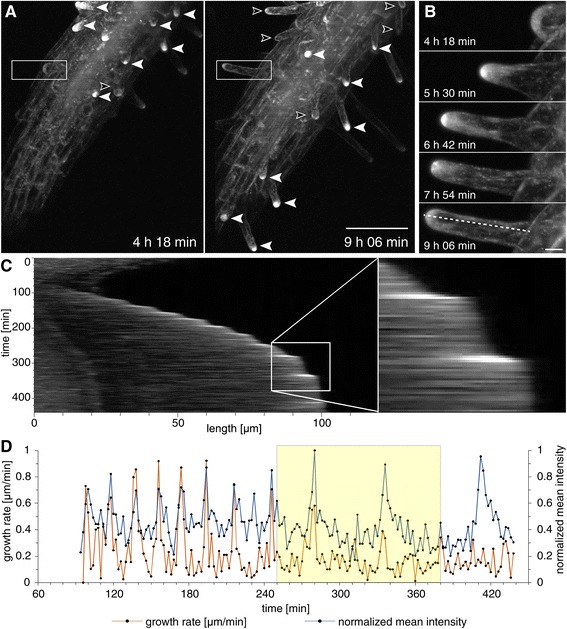
**Light**-**sheet live imaging of GFP**-**RabA1d accumulation during root hair oscillatory tip growth.** Maximum-intensity projections of two indicated time points **(A)**. The actively growing root hairs showed a higher tip-accumulation (filled arrowheads) than the slowly expanding or mature root hairs (open arrowheads). The tip-accumulation of GFP-RabA1d increased in steady state actively growing root hairs (**B**, enlarged boxed area from A). The pixel line from B is plotted as a function of time (kymograph in **C**). The kymograph exhibited an oscillating behavior between lower and higher intensity **(C)**. Fluorescence intensity measurements significantly correlated with the growth rates of the root hair **(D)**. Bars represent 100 μm in A and 10 μm in B.

Root hair growth is a robust example of tip-growth in plant cells, accompanied by polarized membrane trafficking (e.g. [47,48,18]). A previous study demonstrated the localization of RabA1d in the apical dome of growing root hairs [18], however, without following this localization pattern in time. For this reason, we extended the microscopic documentation of GFP-RabA1d in relation to root hair growth kinetics by means of light-sheet microscopy, which allowed following the distribution of GFP-RabA1d for a considerable time encompassing the entire process of root hair growth, from emergence to cessation of elongation. As previously shown [18], GFP-RabA1d labeling was predominantly observed in trichoblast outgrowths (bulges) representing emerging root hairs (Figure 5A,B) and subsequently was largely restricted at the apical dome of vigorously growing root hairs (Figure 5A-C). However, such accumulation of GFP-RabA1d containing vesicles was not visible at the tips of non-growing mature root hairs. In bulges and growing root hairs, GFP-RabA1d colocalized with the endocytotic marker FM4-64 in apical domains (Additional file 1: Figure S7A-F), but in mature root hairs both GFP-RabA1d and FM4-64 were more or less equally distributed through the hair tube (Additional file 1: Figure S7G-I).

Tip-accumulation of GFP-RabA1d was observed in root hair bulges and growing root hairs being progressively reduced as root hair growth rates declined (Figure 5A). The time-lapse imaging showed GFP-RabA1d accumulation in bulge stage which was increased in a time dependent manner (Figure 5B). As shown by fluorescence intensity profiles transverse to the root hair apical dome, GFP-RabA1d signal intensity oscillated between lower and higher intensities which significantly correlated with the pause and growing phases of root hair growth, respectively (Figure 5C,D, Additional file 1: Figure S8A,B). Cross correlation coefficient calculations of root hair growth and GFP-RabA1d intensity for particular periods of time, e.g. between 150–250 min, revealed R^2^ (linear correlation coefficient) values of 0.72, reflecting a strong temporal correlation between apical RabA1d accumulation and tip growth (Figure 5D). The mode of oscillatory growth of root hairs was confirmed also in transgenic *Arabidopsis* line expressing stress-induced mitogen activated protein kinase kinase tagged with YFP (SIMKK-YFP). SIMKK-YFP accumulated in nuclei and cytoplasm of root cells. Observation using light sheet microscopy and determination of fluorescence intensity along transverse profile of growing root hairs showed the oscillatory pattern of root hair growth (Additional file 1: Figure S8C,D, Additional file 4: Video S3). This observation corroborated an assumption that RabA1d participates in oscillatory cell expansion related to intensive and spatio-temporaly controlled vesicle trafficking in root hairs.

### Movement of the vesicular compartments containing GFP-RabA1d depends on the actin cytoskeleton

**Figure 6:**
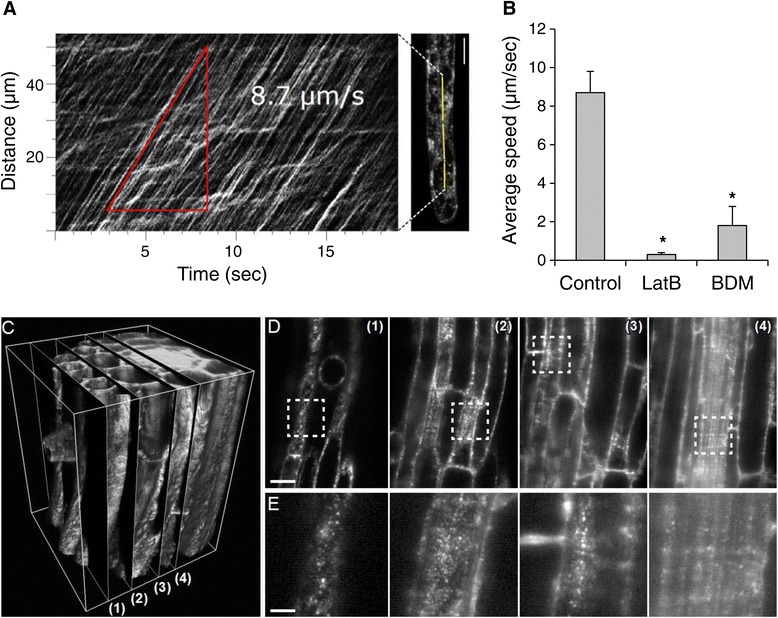
**Motility of GFP**-**RabA1d vesicles in growing root hairs.** Kymograph showing the motility of the GFP-RabA1d vesicles in root hairs **(A)**. Estimation of the average speed of the GFP-RabA1d vesicles in root hairs treated with acto-myosin inhibitors, latrunculin B (LatB) and butane-dione-monoxime (BDM), respectively **(B)**. Mobility and distribution of small RabA1d-positive TGN vesicles in diverse root tissues observed by light-sheet microscopy **(C**-**E)**. 3D reconstruction of a stack of images **(C)**. Single sections at different z-depths ((1): z = 0 μm, (2): z = 16.5 μm, (3): z = 36.8 μm, (4): z = 50 μm) are depicted in **(D)**. The dashed boxed area is enlarged in **(E)**. Bars represent 20 μm in D and 5 μm in E.

The maximum speed of the RabA1d-positive TGN vesicles was measured from kymographs of time-lapse image sequences from root hairs. This analysis revealed that GFP-RabA1d vesicles moved with an average speed of about 8.7 μm/s in root hairs (Figure 6A,B).

Next, *Arabidopsis* seedlings expressing GFP-RabA1d were treated with cytoskeletal inhibitors such as oryzalin (Oryz), latrunculin B (LatB) and butane-dione monoxime (BDM). Previously, we observed that the motility of GFP-RabA1d compartments in trichoblast cells was not affected by oryzalin-induced microtubule disruption by Oryz (Additional file 1: Figure S9A,B). Thus, we used acto-myosin drugs in root hairs to better characterize the movement of GFP-RabA1d compartments along the actin cytoskeleton. Latrunculin B disrupts the actin cytoskeleton by its binding to monomeric G-actin subunits while BDM is affecting myosin by inhibiting its ATPase function. After treatment with latrunculin B, the GFP-RabA1d containing TGN vesicles significantly slowed down and/or stopped their movements through the cytoplasm within 15 min. A similar effect was found after inhibition of vesicle movements using BDM (Figure 6B). These data indicated that motility of the GFP-RabA1d compartments is acto-myosin dependent.

Owing to the extended depth of imaging by means of light sheet microscopy, the high mobility of small RabA1d-positive TGN vesicles was observed in diverse root tissues such as epidermis, cortex, endodermis, pericycle and central cylinder cells. Cells in all these tissues clearly showed presence of the vesicles confirming the role of RabA1d in membrane trafficking in different cells and suitability of light sheet microscopy for deep imaging in thick biological probes (Figure 6C-E, Additional file 5: Video S4).

## Discussion

In this study, we reported the subcellular localization of GFP-tagged RabA1d in transiently and stably transformed plant cells, describing in detail the involvement of RabA1d in active and highly regulated vesicle trafficking events essential for cytokinesis of root meristematic cells and polarized cell expansion of root hairs.

### RabA1d localizes to the early endosome/TGN and is involved in vesicle trafficking

Cellular distribution and localization pattern of GFP-RabA1d found in both transiently transformed leaf cells and stably transformed root cells proved GFP-RabA1d localization to mobile vesicle-like structures, in accordance with the role of the small GTPases in vesicle trafficking. GFP-RabA1d-positive vesicular structures were recognized as TGN compartments, since they were co-labeled with mCherry-VTI12, a SNARE-protein marker which accumulates at the TGN [41,42]. Additionally, GFP-RabA1d vesicles colocalized with FM4-64-positive compartments that are labeled by this vesicular/endocytotic marker soon after entering cells. Merging and co-association at TGN compartment was shown also for other members of RabA class, RabA2, RabA3 and RabA1c which partially overlapped also with the VHA-a1 subunit [24,25]. In TGN compartment, Qa-SNARE group members localize in a complex (SYP41, VTI12, VPS45 and SYP61) [26] and all these compartments are stained by FM4-64 before this dye reaches RabF2 or GNOM- containing compartments [25,26]. Thus, the localization pattern of GFP-RabA1d suggests its specific role during first stages of endocytosis and rapid vesicular trafficking, but not in the process of endosome maturation, because no colocalization with late endosomal markers FYVE-DsRed or mCherry-RabF2a was found. In this respect, the FYVE-domain binds specifically to the phosphoinositol-3-phosphate (PI-3P), which accumulates mainly in the membranes of late endosomal compartments [39]. FYVE domain marker colocalized also with RabF2a and RabF1, today recognized as multivesicular or late endosomal Rab GTPase markers [32,33,40].

The colocalization of GFP-RabA1d with early FM4-64 compartments was confirmed by microscopic analysis. In comparison to RabF2a, another Rab-GTPase that preferentially localize to late/multivesicular endosomes [32-34], GFP-RabA1d colocalized with FM4-64 compartments within minutes upon FM staining. Partial colocalization of YFP-RabF2a with FM4-64 was recorded only later. The BFA treatment of transgenic plants expressing GFP-RabA1d and co-stained with FM4-64 resulted in common relocation and accumulation of both markers in the core of BFA-compartments in root cells. Since it is known that BFA-induced compartments are composed of TGN and plasma membrane-derived endocytotic vesicles in a core, surrounded by remnants of Golgi stacks [44], this localization pattern confirms the residence of RabA1d in early endosome/TGN compartments. Moreover, it is suggestive of the role of RabA1d in the regulation of vesicle recycling, as this process is effectively inhibited by BFA treatment [44,45]. The above suggestion is further supported by the quantitative upregulation of RabA1d after BFA treatment as revealed by proteomic analysis.

The localization and activity of RabA1d at TGN compartment [26] and its responsiveness to BFA suggest a role in vesicle trafficking. Involvement in endocytosis and vesicular recycling has been reported also for other members of RabA class. RabA1c, which is localized at the TGN compartment, is sensitive to endosidin1 but not to wortmannin [24]. Endosidin1 is an inhibitor of endocytosis and vesicular recycling [49], while wortmannin inhibits the transport to vacuoles by affecting pre-vacuolar compartments, TGN vesicles and MVBs [50]. For instance, the effect of endosidin1 on BRI1, PIN2 and AUX1 recycling involves also the mislocalization of early endosome/TGN-compartments, defined by SYP61/VHA-a1 [49]. Similar defects have been observed by the inhibition of vacuolar H^+^-ATPases with concanamycin A (ConcA). ConA blocks the endosome maturation, which leads to production of aggregates of TGN-derived vesicles and therefore, it interferes with the formation of BFA-compartments [26]. The mislocalization of TGN proteins VHA-a1, RabA2a and SYP61 was also found in the *ech* mutant, lacking a TGN-localized ECHIDNA protein crucial for TGN function. This mutant is, however, affected rather in secretion with endocytosis being more or less intact [36]. It raises the question on possible role of RabA1d also in a secretory pathway, which would be parallel to the function of RabA1b in secretory pathway from TGN to the plasma membrane [51]. RabA1b co-localize with VAMP721/722, R-SNARE proteins that operate in the secretory pathway and only partially and unstably associate with TGN, Golgi and endosomes [37]. However, the relocalization of RabA1b to plasma membrane upon wortmannin treatment [37] differs from the accumulation, clustering, fusion or swelling of TGN and MVB compartments, observed by fluorescently tagged RabA1d, RabA4b, RabA1e, VTI12, FYVE and RabF2a [52]. Under wortmannin treatment, RabA1d is relocated into wortmannin-induced multivesicular compartments and is downregulated [52]. Thereby, while the plant counteracts the inhibition of endocytotic recycling by RabA1d upregulation, the inhibition of prevacuolar transport by wortmannin induces RabA1d downregulation [52]. Thus, RabA1d has an important role in vesicle trafficking (endocytotic recycling and probably in the secretory pathway) consistent with its location at early endosome/TGN compartment.

### RabA1d is accumulating in highly active vesicle trafficking zones during cytokinesis and root hair tip-growth

Cell plate arises from the accumulation, fusion and stretching of vesicles directed by centrally-localized complex of cell plate assembly matrix (CPAM). These vesicles are delivered to the growing cell plate by phragmoplast microtubules [53], and cell plate expansion is substantially supported by machineries of both secretory and endocytotic pathways [54,20]. In the growing cell plate, a ring-like structure representing highly active zone of vesicle trafficking is formed [53]. During the initial stages of cell plate formation, GFP-RabA1d accumulated in disc-like structure in the equatorial plane, exhibiting a steady increase of fluorescence intensity likely due to the continuous vesicle fusions. Consistently with the spatial redistribution of active zones of vesicular trafficking during cell plate expansion [53], GFP-RabA1d relocated labeling the rim of the growing cell plates.

The localization of GFP-RabA1d during cytokinesis suggests a role in delivery of cargos and membranes during cell plate formation. It is also compatible with the supporting role of RabA1d in active membrane recycling at cell plate margin domains and consistent with the accumulation of RabA2 and RabA3 subclasses during cell plate formation. Interestingly, they colocalize in the cell plate with the cytokinesis-specific syntaxin KNOLLE (AtSYP111) [25]. Redundant cytokinetic roles have been assigned to three other RabAs, namely RabA1a, RabA1b and RabA1c. These were mislocalized following endosidin1 treatment, while triple knockout mutants (*rab*-*a1a*/*b*/*c*) were hypersensitive to endosidin1 induced cytokinetic disruption [24]. Similarly, the cytokinetic-defective mutant *pas2* (mutant of the microsomal elongase gene PASTICCINO2), showed delayed and defective cell plate formation due to the heterogeneous aggregation of RabA2a and KNOLLE, while RabF2a did not show any defect. Thus, early endosomes/TGN compartments play a specific role during cell plate formation [55]. The transport between TGN and the plasma membrane by RabA1b interacting with VAMP721 [37] is involved in cytokinesis and the inhibition of trafficking at TGN by ConA affects cell plate formation through the mislocalization of early endosomes/TGN compartments [56]. Additionally, the colocalization of RabA1d with VTI12, a member of SNARE complex, suggests a role also in the transport to the plasma membrane [57]. Thereby, the role of RabA1d during cell plate formation is consistent with the proposed activity of early endosome/TGN compartments in the coordination of secretory, endocytotic and recycling pathways.

Similarly to cytokinesis, RabA1d participates in localized cell expansion where increased vesicle trafficking is required. The tip-accumulation of RabA1d in root hair bulges and in growing root hairs confirmed the role of some RabAs during polar growth. The disruption of RabA4d in pollen tubes reduced cell polarity, expansion and displayed altered deposition of cell wall components [15]. RabA4b showed tip-localization and regulated root hair tip growth through a compartment involved in the polarized secretion, and similarly to RabA1d, the tip-localized accumulation disappears in mature root hairs or after latrunculin B treatment [16]. The RabA2 silencing led to reduction in number and length of the root hairs, showing an important role not only in expansion but also during root hair initiation [17].

The RabA1d accumulation in bulge stage was increased during the progression to the tip growth. In growing root hairs, GFP-RabA1d signal intensity fluctuated in oscillatory manner. Oscillations between lower and higher intensity labeling of GFP-RabA1d at the root hair tip temporally coincided with the pause and growth phases of root hair tip growth, respectively. Similarly to pollen tubes [58], root hairs exhibit oscillatory growth along with other events accompanying tip growth like cell wall modifications, cytoskeleton rearrangements and targeted vesicle trafficking [59]. Some factors, such as Ca^2+^ gradient [60], extracellular pH and ROS concentration [61] and actin organization [62,63] have been found to be associated with the oscillatory character of tip growth and are closely related to vesicle trafficking. Consistently with the frequency of vesicular trafficking during root hair initiation and growth, GFP-RabA1d accumulation was lower at bulge stage than in growing root hairs. After the local structural changes that are required to define the bulge position, such as cytoskeleton rearrangements [64], cell wall composition [65,66] and accumulation of structural sterols in the plasma membrane [18], an increased vesicle trafficking is required during root hair elongation to provide a new plasma membrane in the expanding zone. This zone is filled with secretory and endocytotic vesicles [2], highly dynamic early endosomes in the clear zone and larger endosomal compartments in the subapical region [67]. Thereby, RabA1d is localized at the tip-zone of growing root hair as previously reported [18] and its accumulation is spatio-temporally correlated with the root hair tip expansion and vesicle trafficking.

### High motility of TGN vesicles containing GFP-RabA1d depends on the actin cytoskeleton

Endosomes show different patterns of motility including stationary phases, slow or rapid movements [40]. The microtubule disruption by oryzalin did not affect the motility of RabA1d-positive vesicles, however the acto-myosin inhibitors affected extremely their motility. Similarly, the tip-localization of RabA4b was affected by latrunculin B treatment but not by oryzalin [16]. It suggests an important role of actin cytoskeleton in RabA1d motility, however, the organization and dynamics of several endosomal structures is influenced by both actin and microtubule cytoskeletons [40,68,69] and their motor proteins, i.e. myosins [70]. The role of the actin cytoskeleton in vesicular trafficking was strengthened by proteomic study after BFA treatment, which showed significant upregulation of profilin 2 (an actin binding protein) and its accumulation in BFA-compartments [71]. Finally, vigorous motility of RabA1d-containing TGN vesicles was also observed in diverse root tissues such as epidermis, cortex, endodermis, pericycle and central cylinder cells confirming the role of RabA1d in vesicular trafficking in different cells and not only associated to tip-growth.

## Conclusions

We show that RabA1d colocalized with early FM4-64 endocytotic compartments and with VTI12, a TGN marker. BFA treatment not only induced RabA1d upregulation but also relocalization of GFP-RabA1d to the core of BFA-compartments. Most importantly, a specific spatio-temporal accumulation of RabA1d correlated with vesicle trafficking requirements during both cell plate formation and root hair oscillatory tip growth.

## Methods

### Cloning of *35S*::*GFP*:*RabA1d* construct

The coding sequence of *RabA1d* (At4g18800) gene was PCR-amplified from Columbia-0 cDNA using the following primers: Forward: 5′-GCG GAT CCG TGT TAA TGG CGG GTT-3′, Reverse: 5′-GCG GAT CCT TTA GGA CAT AAG ACC AT-3′. The indicated BamHI restriction sites were used to ligate the coding sequence into the *pCATgfp*-vector. The resulting *35S*::*GFP*:*RabA1d* expression cassette was excised by HindIII and ligated into the binary vector *pCB302* [72]. The construct was used to transform competent GV3101 (pMP90) *Agrobacterium tumefaciens* cells.

### Plant transformation

Epidermis cells of *Allium porrum* were transiently transformed with *35S*::*GFP*:*RabA1d* construct by particle bombardment [73] with the Biolistic PDS-1000/He system (Bio-Rad Laboratories GmbH, München, Germany). Leaves of *Nicotiana benthamiana* were transiently transformed by infiltration with *Agrobacterium tumefaciens* [74]. The bacterial optical density (OD_600_) was 0.2 for all experiments. For co-expression, bacterial suspensions were combined shortly before infiltration. *35S*::*GFP*:*RabA1d* construct was co-expressed with *35S*::*ER*:*DsRed* to visualize the ER cortical network [38]; *pUBQ10*::*mCherry*:*VTI12*, a SNARE-protein which is known to accumulate at the TGN [41,42]; *35S*::*2xFYVE*:*DsRed* [39,40] and *pUBQ10*::*RabF2a*:*mCherry* [4], for late endosomes in leaf cells of *Nicotiana benthamiana. Arabidopsis thaliana* plants of the ecotype Columbia-0 were transformed by the *Agrobacterium tumefaciens*-mediated floral dip method [75]. Stable transformed plants were selected by kanamycin resistance. Expression of SIMKK-YFP was documented in stably transformed plants of Arabidopsis [76].

### Immunoblotting

Protein extract samples were precipitated according to the method described by Wessel and Fluegge [77]. Proteins were separated on SDS PAGE gels and transferred to a polyvinylidene difluoride membrane in a wet tank using transfer buffer for 1.5 h [78]. For immunodetection of proteins, the membrane was blocked with 6% (w/v) BSA in Tris-buffered saline (TBS) buffer for 1 h, and subsequently incubated with anti-GFP antibody (Roche) diluted 1:300 in TBS-T buffer (TBS 0.1%, Tween 20) containing 1% (w/v) BSA for 1.5 h at room temperature or at 4°C overnight. After washing in TBS-T the membrane was incubated with secondary antibody, goat anti-mouse IgG, AP Conjugate (Molecular Probes) diluted 1:500 in TBS-T containing 1% (w/v) BSA at room temperature for 1.5 h. Following several washing steps, proteins were detected by incubating the membrane in freshly prepared ECL reagent (GE Healthcare) for 2 min. Luminescence was detected using Hyperfilm ECL in a dark room (GE Healthcare).

### FM4-64 staining and BFA treatment

To visualize early endosomes, seedlings were mounted during 10–15 min in culture medium with FM4-64, used in the concentration of 5 μg/ml. For colocalization study, seedlings were placed on microscopic slide into drop of FM4-64, covered by coverslip and transferred immediately to the microscope. Root cells were recorded every 5 min. For BFA treatment, seedlings were incubated in culture medium containing 50 μM brefeldin A (BFA). For washout experiments, seedlings were incubated in 50 μM BFA and 5 μM FM4-64 for 30 min, washed thoroughly on slide by culture medium for 5 min and observed in the microscope for additional 60 min.

### Proteomic analysis

The proteomic analysis was done following the methodology previously described [71]. *Arabidopsis thaliana* L. seedlings (ecotype Columbia) were grown during 10 days. Seedlings were surface-treated for 2 hours with liquid ½ MS-media containing 50 μM BFA or mock solution containing same final concentration of DMSO. Roots were quickly dissected and homogenized to a fine powder using mortar and pestle in liquid nitrogen. Total protein was extracted using phenol method as previously described [79].

### Two-dimensional electrophoresis

The extraction of the protein samples from the pellet and two-dimensional electrophoresis was done following the method previously described [71]. The gels were stained by Bio-Safe coomassie brilliant blue staining solution (Bio-Rad), scanned using a densitometer (GS-800, Bio-Rad) and analyzed using the software package PD-Quest 8.0 (Bio-Rad) as described by Takáč et al. [71].

### In gel trypsin digestion and mass spectrometry

The trypsin digestion process and peptide mix extraction was performed as described previously [71], it was spotted onto an anchor chip target (Bruker Daltonics) using the dried droplet method [80]. α-Cyano-4-hydroxycinnamic acid (2 mg/ml in 50% (v/v) ACN containing 0.2% (v/v) trifluoroacetic acid (TFA)) was used as energy absorbing molecule (matrix). Mass spectrometry analysis was performed using a MALDI-TOF-TOF (Ultraflex II, Bruker Daltonics) using the acquisition settings described by Takáč et al. [71], similarly as the data processing using the Bruker software packages Flex analysis 2.4 and BioTools 3.1, the Mascot search (Mascot Server 2.2.03), and the data matching in Swissprot database version 54.6, containing 29315 entries. For protein/peptide identification, standard scoring and a significance threshold of P < 0.05 were chosen as Mascot result parameters.

### Microscopic analysis

The microscopic analysis was performed using an Olympus FV1000 upright confocal laser scanning microscope (with a 63x/NA 1.3 oil immersion objective), and a Zeiss LSM 710 (with a 63x/NA 1.4 oil immersion objective). For co-expression studies, the images were acquired using the sequential line-scanning mode to avoid bleed through. The colocalization analysis was done using ImageJ toolbox JACoP, Pearson’s coefficient (r) was determined after Costes automatic threshold [81].

For time-lapse observations we used the Andor Revolution xD confocal spinning disc system (Andor Technology, Belfast, Ireland) combined with the Olympus inverted IX71 microscope and the Olympus UPLANSAPO 60xW/1.2 objective, and Zeiss confocal spinning disc system combined with a Yokogawa CSU-X1 scanning head, equipped with a 63x/NA 1.4 oil immersion objective. Single focal planes were recorded with 20 frames per second.

Speed measurements of single endosomes were extracted from acquisitions of 50 root hairs using the Multiple Kymograph plugin for ImageJ software (http://rsbweb.nih.gov/ij/). A kymograph is a two dimensional (2-D) graphical representation of a three dimensional (3-D) image sequence (x, y, time). The movement of a particle along a defined 1-D line is displayed over time (y, time). The 1-D pixel line is cut out in every frame of the video sequence and displayed side by side. A particle moving along this line will appear as a diagonal stripe in the kymograph and its slope equals the speed of the particle.

Light-sheet microscopy was performed in custom built system as previously described [46] or with light sheet fluorescence microscope Z.1 from Zeiss. In the custom system seven days old *Arabidopsis* seedlings were imaged every two minutes for a period of up to 10 h using a Carl Zeiss N-Achroplan 40x/0.75 objective lens in the detection path and a Carl Zeiss Plan-Neofluar 5x/0.16 objective lens in the illumination path. Each stack of images contains 388 planes spaced 0.645 μm along the z-axis and was recorded with the Andor NEO camera (Andor Technology, Belfast, Ireland). Z.1 light sheet fluorescence microscope system was equipped with a Carl Zeiss W Plan-apochromat 20x/1.0 detection objective and a Carl Zeiss LSFM 10x/0.2 illumination objective. Images were recorded with the PCO.Edge sCMOS camera (PCO AG, Kelheim, Germany).

Cell plate growth rate was obtained by dividing cell plate diameter with the total time required for full cell plate expansion (i.e., until the cell plate rim reached the parent walls) in root meristematic zone of seedlings expressing GFP-Rab1Ad and GFP-MAP4 as control. In both cases, more than 10 cells were measured and average growth rate was calculated. Only complete cell plate formations were taken into account.

For root hair growth and fluorescence intensity measurements the images were cropped to smaller regions. The mini-stack was rotated for a side view on the root hair. After a background subtraction step, the image stack was flattened using a sum-projection. These image sequences were used to track the root hair tip for growth rate and fluorescence intensity was estimated using Adobe After Effects.

## Additional files

Additional file 1: Figure S1. Subcellular localization of GFP-RabA1d in diverse plants. **Figure S2.** FM4-64 uptake and colocalization with early and late endosomes. **Figure S3.** Redistribution of FM4-64-positive and GFP-RabA1d-positive compartments in BFA-treated root epidermal cells after BFA washout. **Figure S4.** RabA1d identification in the proteomic analysis. **Figure S5.** Accumulation and redistribution of GFP-RabA1d during cell plate initiation. **Figure S6.** The effect of *35S*::*GFP*:*RabA1d* overexpression on plant growth in transgenic *Arabidopsis* plants. **Figure S7.** Colocalization of GFP-RabA1d and FM4-64 in *Arabidopsis* root hairs. **Figure S8.** Root hair oscillatory tip growth in seedlings expressing GFP-RabA1d and SIMKK-YFP. **Figure S9.** Effect of Oryzalin (Oryz) on the motility of GFP-RabA1d compartments. Additional file 2: Video S1. Localization of GFP-RabA1d during the cell plate formation in cytokinetic cells. Additional file 3: Video S2. Cell plate formation in Arabidopsis primary root expressing GFP-MAP4. Maximum intensity projections of Z-stacks collected every 1 min for a time period of 60 minutes. Scale bar: 20 μm. Additional file 4: Video S3. Root hair tip growth in Arabidopsis plants expressing SIMKK-YFP observed by light sheet microscopy. Video sequence of a root hair growing over a time period of 1 h, 15 min and 1 s. Additional file 5: Video S4. Deep imaging and mobility of small RabA1d-positive TGN vesicles in diverse root tissues observed by light sheet microscopy.
