# Supplementary material for: Trans-Golgi network localized small GTPase RabA1d is involved in cell plate formation and oscillatory root hair growth
Source: BMC Plant Biol. 2014 Sep 27;14:252. doi: 10.1186/s12870-014-0252-0 (PMC4180857; doi:10.1186/s12870-014-0252-0)
Supplement: Additional file 1: Figure S1. — Subcellular localization of GFP-RabA1d in diverse plants. Figure S2. FM4-64 uptake and colocalization with early and late endosomes. Figure S3. Redistribution of FM4-64-positive and GFP-RabA1d-positive compartments in BFA-treated root epidermal cells after BFA washout. Figure S4. RabA1d identification in the proteomic analysis. Figure S5. Accumulation and redistribution of GFP-RabA1d during cell plate initiation. Figure S6. The effect of 35S::GFP:RabA1d overexpression on plant growth in transgenic Arabidopsis plants. Figure S7. Colocalization of GFP-RabA1d and FM4-64 in Arabidopsis root hairs. Figure S8. Root hair oscillatory tip growth in seedlings expressing GFP-RabA1d and SIMKK-YFP. Figure S9. Effect of Oryzalin (Oryz) on the motility of GFP-RabA1d compartments. [file 12870_2014_252_MOESM1_ESM.pdf]

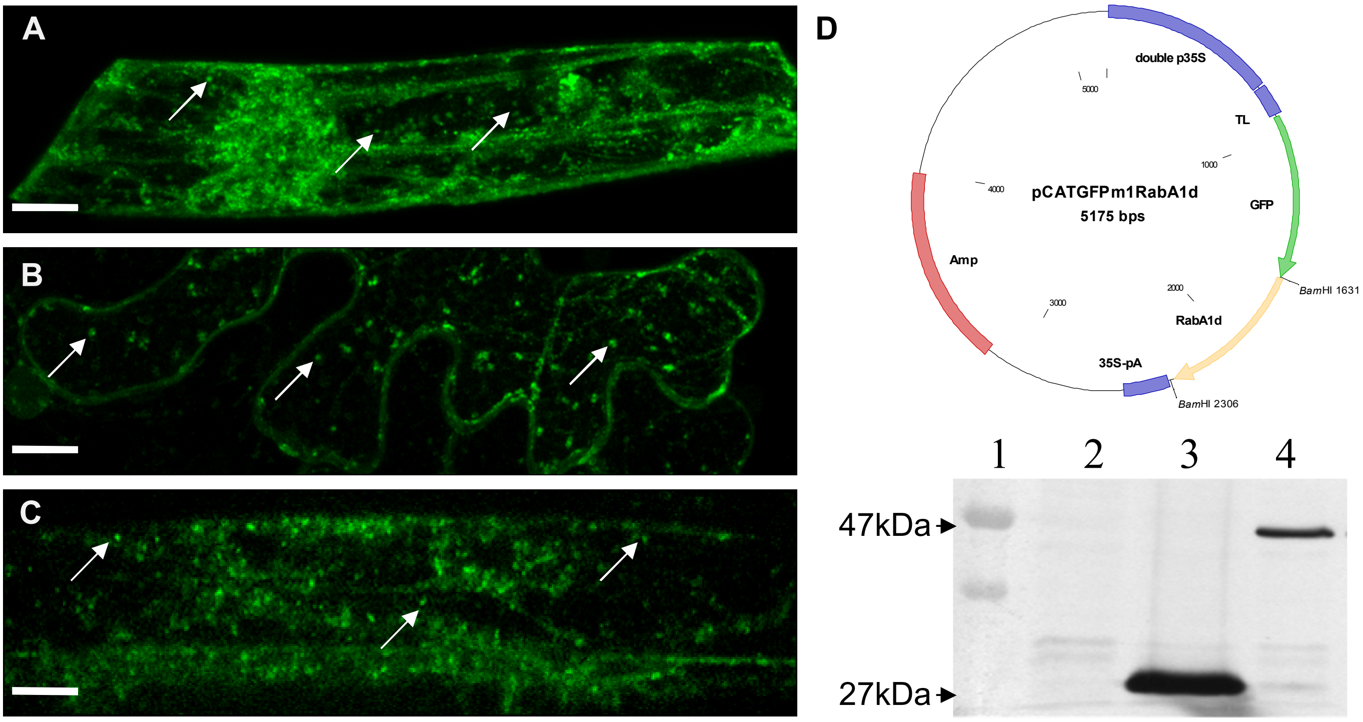

Figure S1. Subcellular localization of GFP-RabA1d in diverse plants. In transiently transformed *A. porrum* (**A**) and *N. benthamiana* (**B**) leaves and rhizodermal cells of stably transformed *A. thaliana* (**C**), GFP-RabA1d was always localized to vesicle-like structures which moved rapidly in the cytoplasm. *35S*::*GFP*:*RabA1d* construct and immunoblot analysis using GFP-specific antibody in stably transformed *Arabidopsis* plants (**D**). In lane 1 is the standard with molecular weight marker, followed by non-transgenic Col-0 in lane 2, GFP specific band (29 kDa) from GFP stably expressed in *Arabidopsis* line is in lane 3 and the specific band of 46 kDa representing GFP-RabA1d in stably transformed plants is in lane 4. Slightly labeled additional bands in control and transgenic lines are non-specific and might result from weak non-specific binding of secondary antibody. Bars represent 8 μm in A-C.

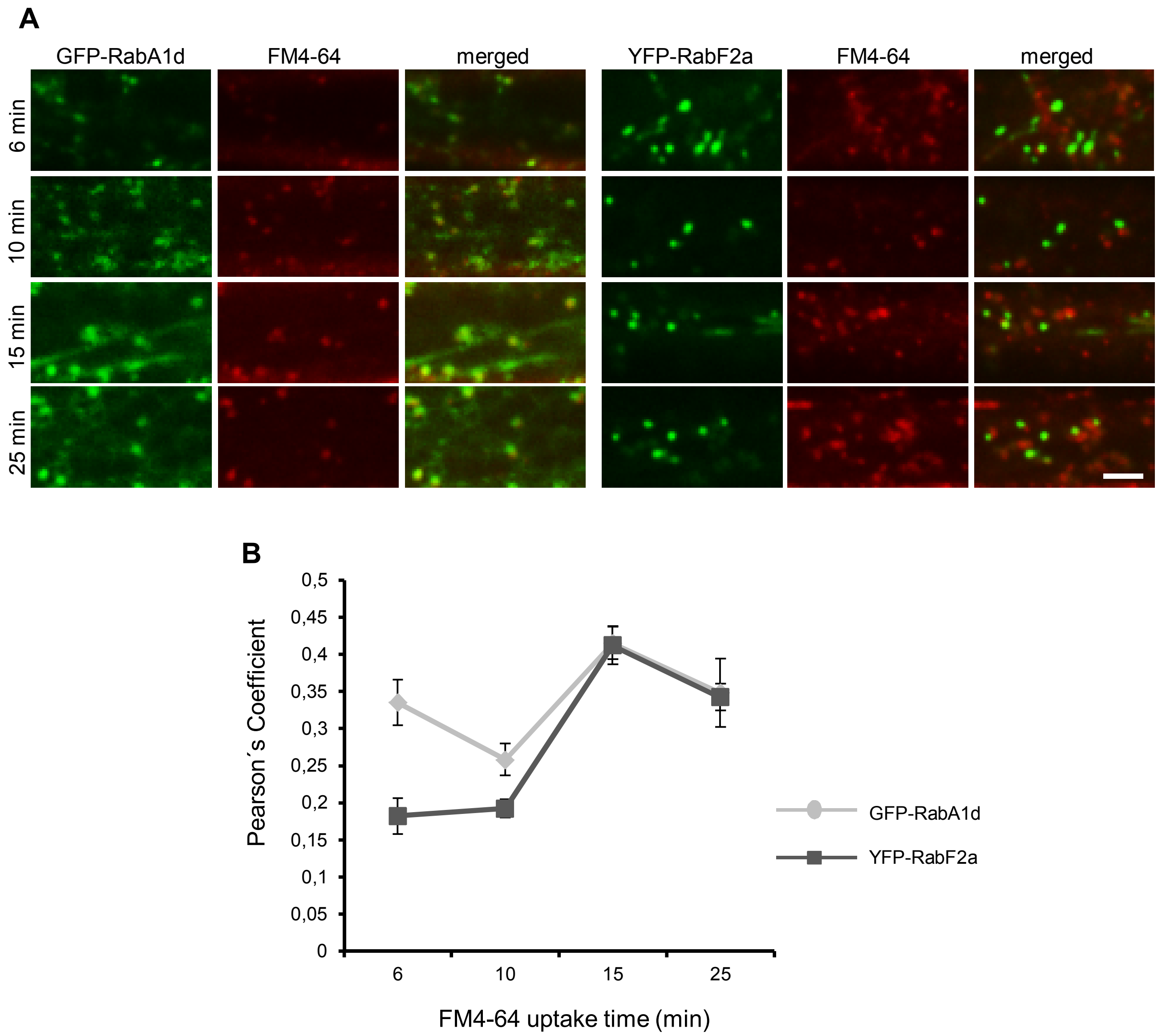

Figure S2. FM4-64 uptake and colocalization with early and late endosomes. FM4-64 compartments colocalizing with GFP-RabA1d and YFP-RabF2a after 6, 10, 15, 25 min uptake (**A**). Early FM4-64 compartments colocalize with GFP-RabA1d after 6 min, while late endosomes visualized with YFP-RabF2a, colocalize after 15 min. Pearson´s coefficient of GFP-RabA1d and YFP-RabF2a with FM4-64 compartments (**B**). Pearson´s coefficient was determined using Costes automatic threshold. Bar represents 4 µm in A. Error bars represent SE.

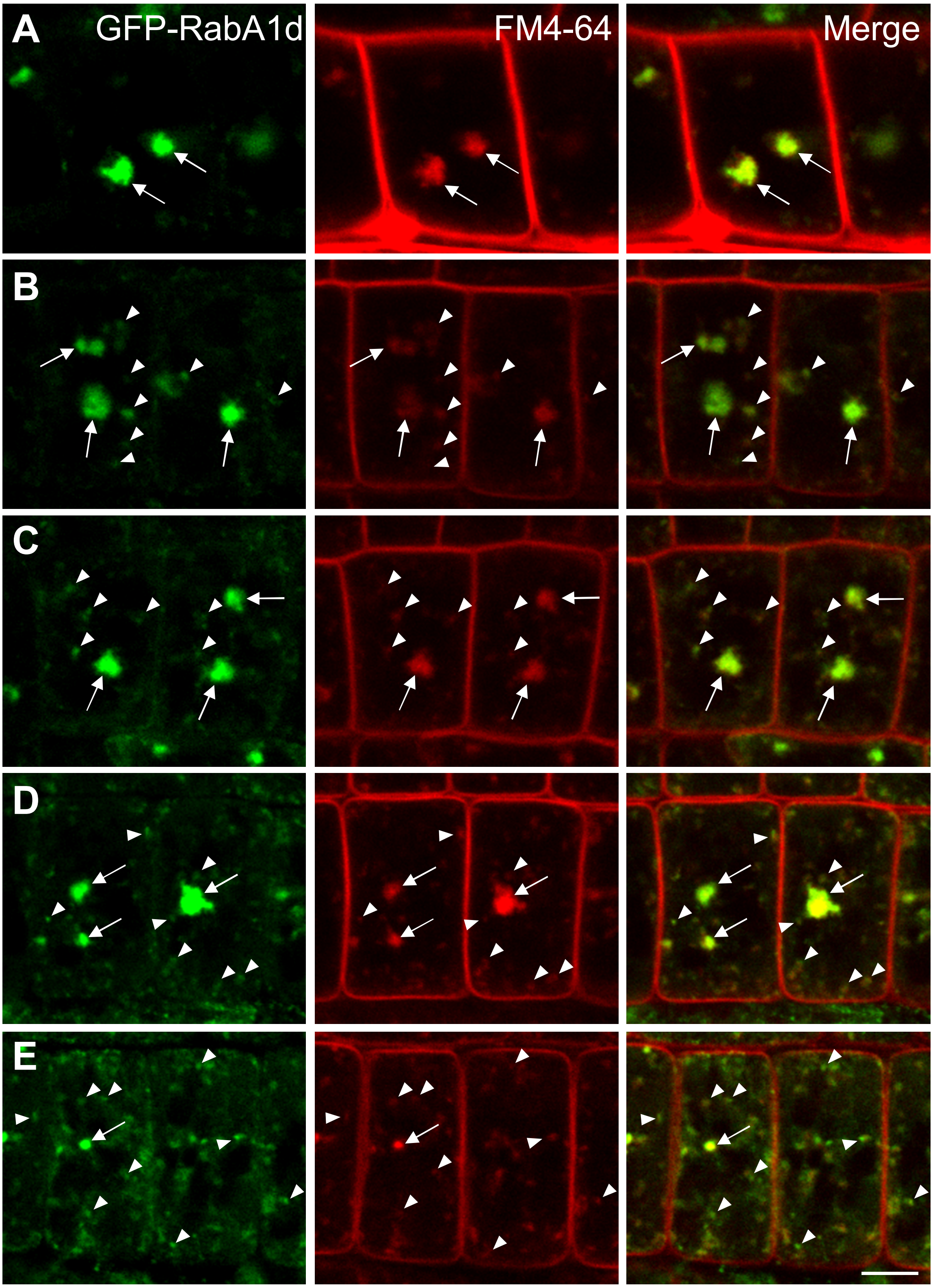

Figure S3. Redistribution of FM4-64-positive and GFP-RabA1d-positive compartments in BFA-treated root epidermal cells after BFA washout. Roots were treated with BFA together with FM4-64 for 30 min (**A**) and after washout localization of both FM4-64 and GFP-RabA1d was followed at 5 min (**B**), 10 min (**C**), 30 min (**D**) and 60 min (**E**). BFA compartments were gradually disintegrated and release of GFP-RabA1d-positive compartments that co-localized with FM4-64 was apparent already after 5 min (**B**). Bar represents 5 μm.

A

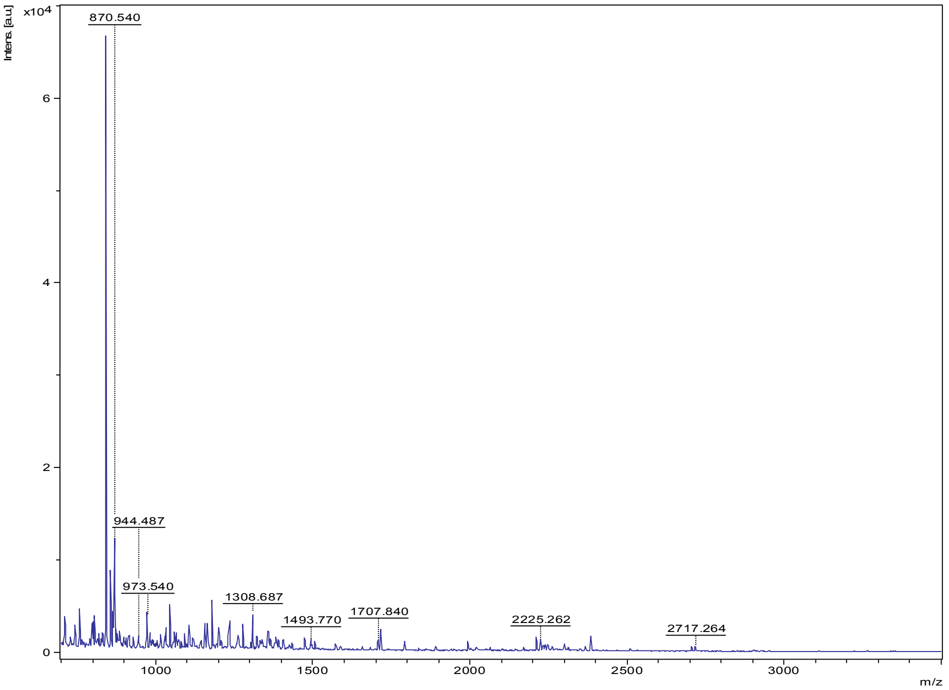

B

MATRIX SCIENCE Mascot Search Results

```
User            : BioTools Batch
Email           :
Search title    : Auto submitted by BioTools
Database        : A.thaliana thaliana_92 (28159 sequences; 12091518 residues)
Timestamp       : 31 May 2007 at 07:55:19 GMT
Top Score       : 60 for ath:At4g18800,  F28A21.210; Ras-related GTP-binding family protein
```

Probability Based Mowse Score

Protein score is -10*Log(P), where P is the probability that the observed match is a random event.
Protein scores greater than 57 are significant (p<0.05).

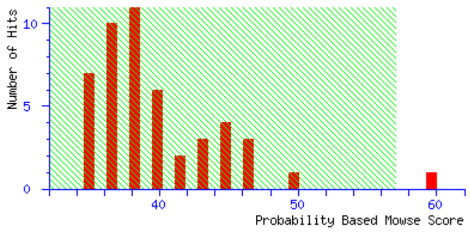

Concise Protein Summary Report

Format As	Concise Protein Summary	Help
	Significance threshold p< 0.05	Max. number of hits AUTO

Re-Search All | Search Unmatched

```
1.     ath:At4g18800     Mass: 23969     Score: 60     Expect: 0.031   Queries matched: 7
       F28A21.210; Ras-related GTP-binding family protein

2.     ath:At2g01900     Mass: 48407     Score: 50     Expect: 0.28   Queries matched: 9
       T23K3.9; endonuclease/exonuclease/phosphatase family protein
```

Search Parameters

```
Type of search          : Peptide Mass Fingerprint
Enzyme                  : Trypsin
Fixed modifications     : Carbamidomethyl (C)
Mass values             : Monoisotopic
Protein Mass            : Unrestricted
Peptide Mass Tolerance  : ± 70 ppm
Peptide Charge State    : 1+
Max Missed Cleavages    : 1
Number of queries       : 62
```

Figure S4. RabA1d identification in the proteomic analysis. Mass spectrometry analysis was performed using a MALDI-TOF-TOF (**A**). Mascot results from protein/peptide identification using standard scoring and a significance threshold of $P < 0.05$ (**B**).

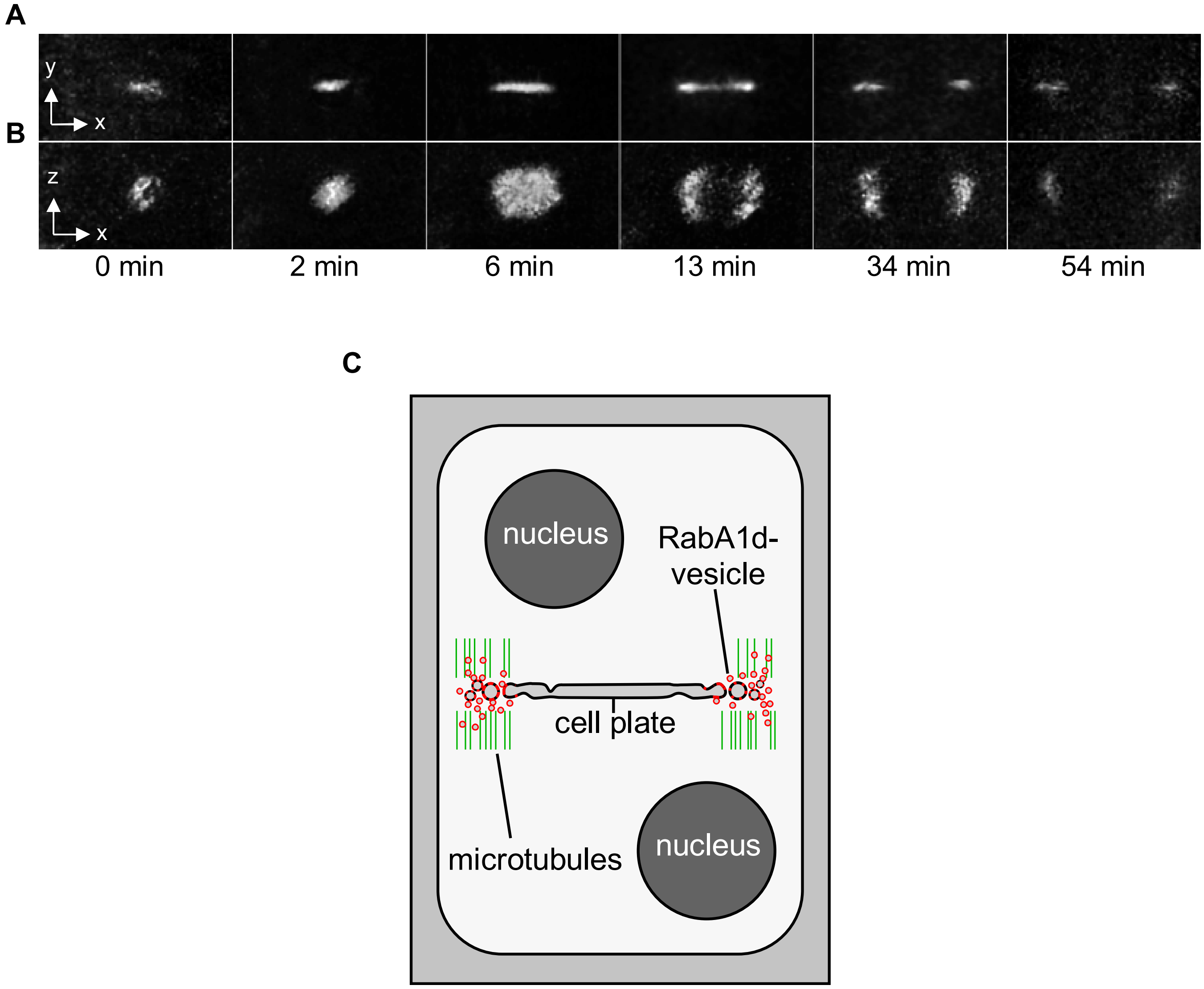

Figure S5. Accumulation and redistribution of GFP-RabA1d during cell plate initiation. GFP-RabA1d accumulation during cell plate initiation and growth observed by real time spinning disc microscopy at indicated time points, side (**A**) and top (**B**) view. Proposed model of the GFP-RabA1d accumulation at the margins of growing cell plate (**C**), the RabA1d-positive vesicles are colored in red.

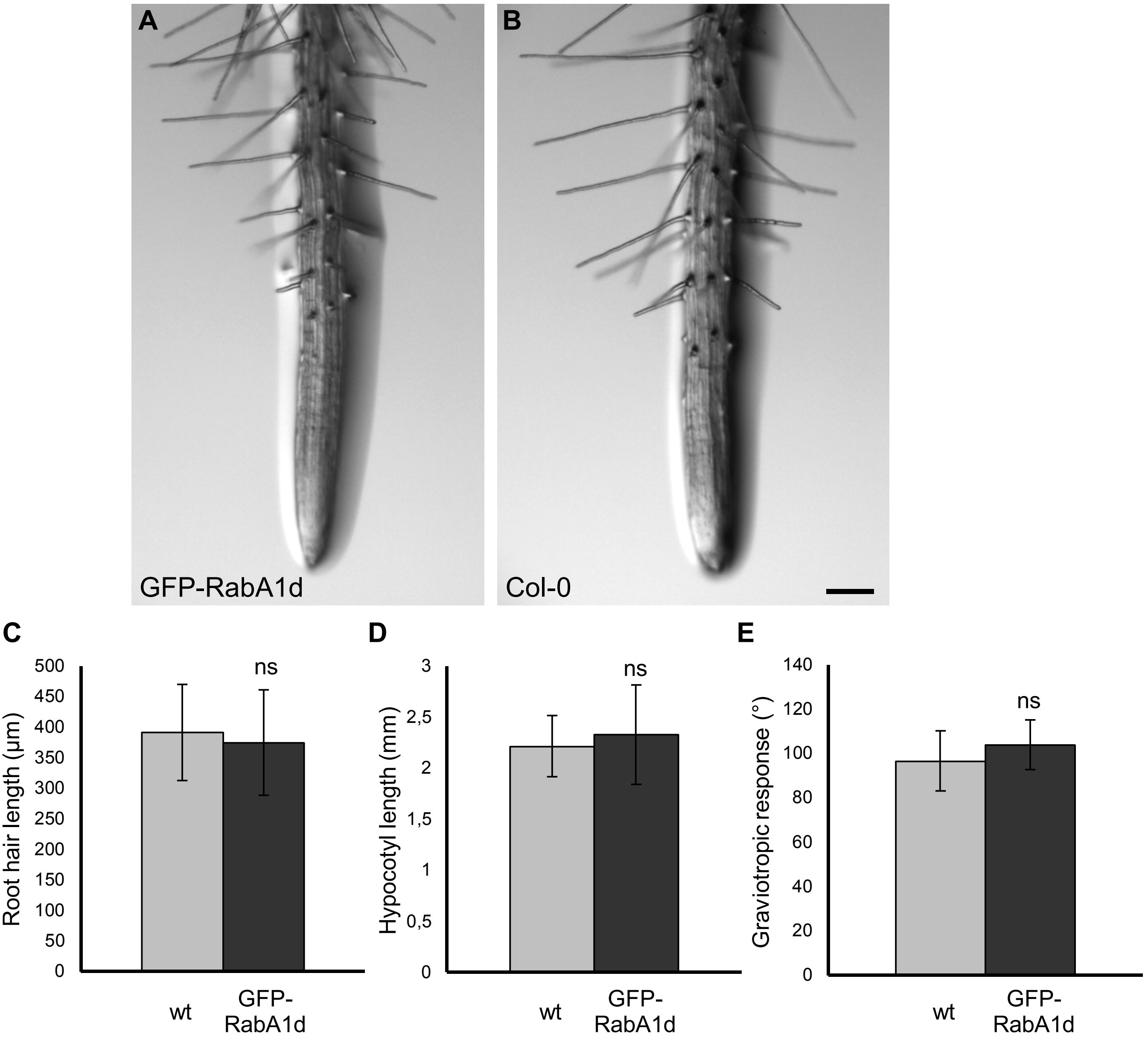

Figure S6. The effect of *35S*::*GFP*:*RabA1d* overexpression on plant growth in transgenic *Arabidopsis* plants. Root tip with developing root hairs of young seedlings of Col-0 (**A**) and GFP-RabA1d (**B**) 24 h after germination. Graphs showing that overexpression by 35S::*GFP*:*RabA1d* construct in *Arabidopsis* have no negative effect on the growth of seedling root hairs and hypocotyls as well as on the gravitropic root response (**C**-**E**). Error bars represent SD, ns: no significant differences, t-test $p < 0.01$. Bar represents 100 µm in A and B.

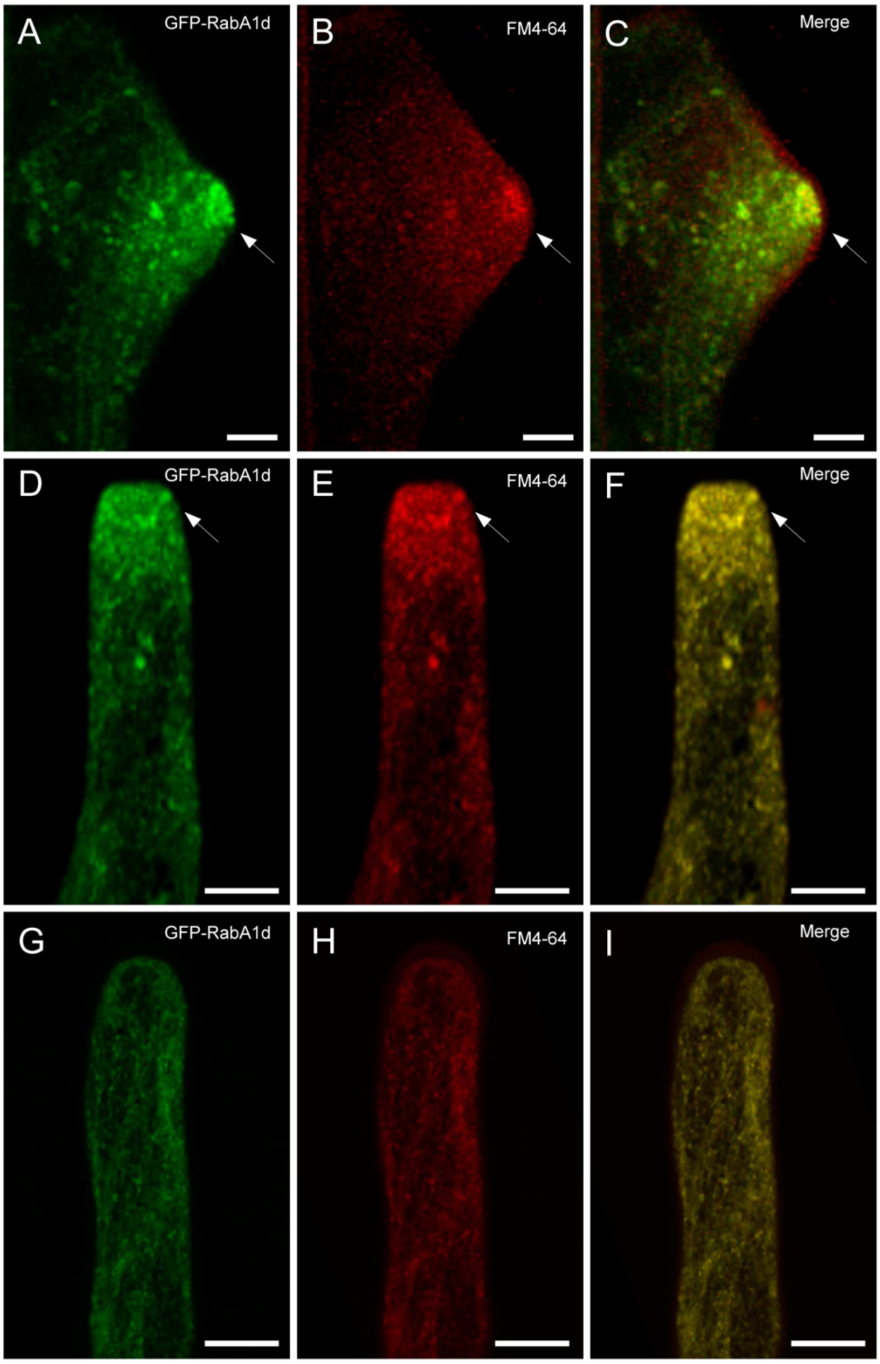

Figure S7. Colocalization of GFP-RabA1d and FM4-64 in *Arabidopsis* root hairs. GFP-RabA1d colocalized and accumulated together with FM4-64 in root hair bulges (**A**-**C**) and at the tip of growing root hairs (**D**-**F**). Colocalization but no accumulation at the tip was recorded in the non-growing mature root hairs (**G**-**I**). Bars represent 5 µm in A-C and 7 µm in D-I.

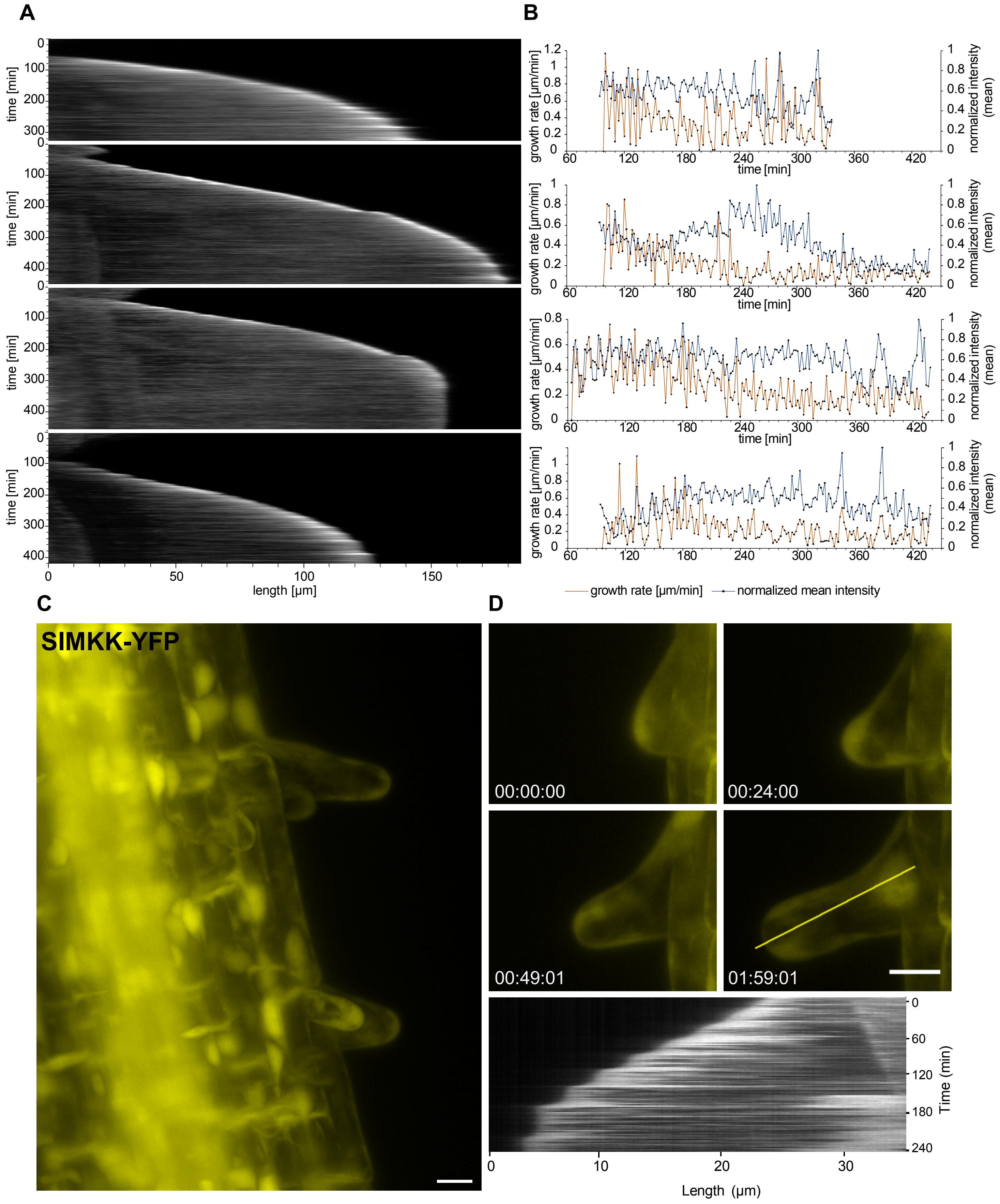

Figure S8. Root hair oscillatory tip growth in seedlings expressing GFP-RabA1d and SIMKK-YFP. GFP-RabA1d accumulation during root hair oscillatory tip growth in four root hairs of the data set presented in figure 5 (**A**-**D**). Kymographs (**A**) and measurements (**B**) show the significant correlation of the mean intensity fluorescence with the growth rates in each root hair. Maximum-intensity projections of SIMKK-YFP expression in root cells and root hairs observed by light sheet microscopy (**C**). Stages of root hair tip growth in seedlings expressing SIMKK-YFP at indicated time points and kymograph exhibiting an oscillatory pattern of root hair tip growth (**D**). Bars represent 10 µm.

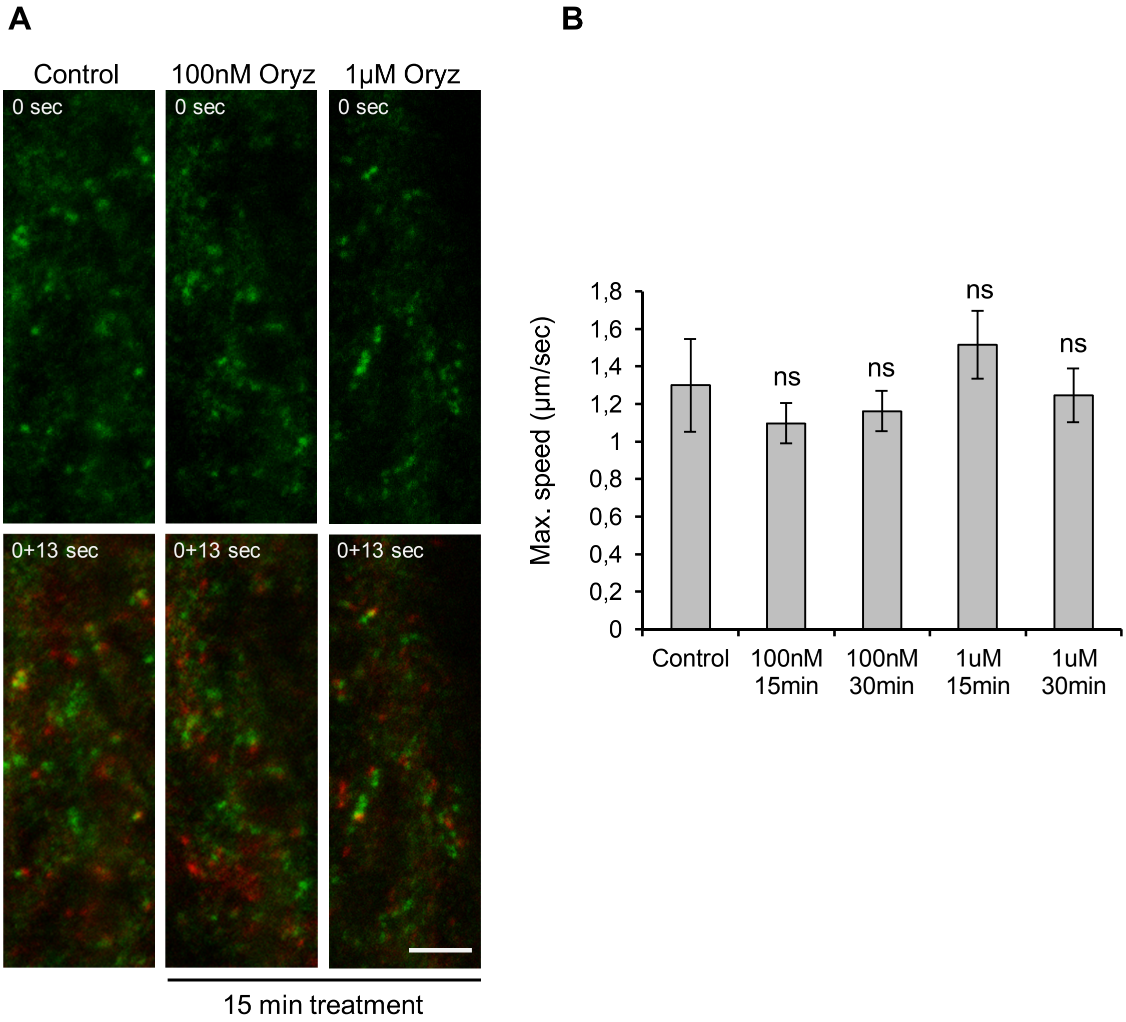

Figure S9. Effect of Oryzalin (Oryz) on the motility of GFP-RabA1d compartments. Trichoblast section showing the comparable mobility in control and oryzalin treated cells at 100 nM and 1 µM concentration (**A**). Merged image of 0 and 13 seconds time point show the high mobility of endosomes, few endosomes remained in same position during the time. Maximal speed of GFP-RabA1d compartments after 15 and 30 min treatment with oryzalin at 100 nM and 1 µM concentration (**B**). Error bars represent SE, ns: no significant differences, t-test $p < 0.01$. Bar represents 5 µm in A.
